# Is Uric Acid a Missing Link between Previous Gestational Diabetes Mellitus and the Development of Type 2 Diabetes at a Later Time of Life?

**DOI:** 10.1371/journal.pone.0154921

**Published:** 2016-05-11

**Authors:** Piotr Molęda, Aneta Fronczyk, Krzysztof Safranow, Lilianna Majkowska

**Affiliations:** 1 Department of Diabetology and Internal Medicine, Pomeranian Medical University in Szczecin, Szczecin, Poland; 2 Department of Biochemistry and Medical Chemistry, Pomeranian Medical University in Szczecin, Szczecin, Poland; Xinhua Hospital, Shanghai Jiaotong University School of Medicine, CHINA

## Abstract

**Introduction:**

A high level of uric acid (UA) is a strong, independent risk factor for type 2 diabetes mellitus. The relationship between UA levels and the development of type 2 diabetes in women with previous gestational diabetes mellitus (pGDM) remains unclear. The aim of study was to evaluate the UA levels in pGDM women in relation to their current nutritional status and carbohydrate metabolism.

**Material and Methods:**

199 women with pGDM diagnoses based on oral glucose tolerance tests (OGTTs) 5–12 years previously and a control group of 50 women without pGDM. The assessment included anthropometric parameters, body composition (Tanita SC-330S), current OGTT, insulin resistance index (HOMA-IR), β-cell function (HOMA-%B), HbA1c, lipids, and uric acid.

**Results:**

No differences between groups were found in terms of age, time from the index pregnancy, anthropometric parameters, lipids or creatinine levels. The incidences of overweight and obesity were similar. Carbohydrate abnormalities were more frequent in the pGDM group than the control group (43.2% vs 12.0% p<0.001). The women with pGDM had significantly higher fasting glucose, HbA1c, glucose and insulin levels in the OGTTs, but similar HOMA-IR values. Their UA levels were significantly higher (258±58 vs 230±50 μmol/L, p<0.005) and correlated with BMI and the severity of carbohydrate disorders. The normal weight and normoglycemic pGDM women also demonstrated higher UA levels than a similar control subgroup (232±48 vs 208±48 μmol/L, p<0.05). Multivariate analysis revealed significant correlations of UA level with BMI (β = 0.38, 95% CI 0.25–0.51, p<0.0001), creatinine level (β = 0.23, 95% CI 0.11–0.35, p<0.0005), triglycerides (β = 0.20, 95% CI 0.07–0.33, p<0.005) and family history of diabetes (β = 0.13, 95% CI 0.01–0.25, p<0.05). In logistic regression analysis, the association between higher UA level (defined as value ≥297 μmol/L) and presence of any carbohydrate metabolism disorder (IFG, IGT or diabetes) was statistically significant (odds ratio 3.62 [95% CI 1.8–7.3], p<0.001).

**Conclusions:**

Higher UA levels may be associated with the development of type 2 diabetes in pGDM women, also in these with normal body weights.

## Introduction

Increased levels of uric acid (UA) often coexist with obesity, type 2 diabetes mellitus, hypertension and atherosclerosis [1–3]. High UA levels have been recognized as one of the markers of metabolic syndrome and increased cardiovascular risk [4,5]. Although the increase of UA is believed to be associated with insulin resistance, it remains unclear whether, it is secondary to insulin resistance or a primary disorder that is involved in its development.

Prospective studies that have been performed in different populations, i.e., European, American and Chinese, have indicated that elevated serum UA levels are a strong, independent risk factor for type 2 diabetes mellitus [6–8], and this finding has been confirmed in recently published systematic reviews and meta-analyses [9–11]. As indicated by the findings of these cited studies, higher concentrations of uric acid increase the risk of developing diabetes regardless of the presence of other risk factors, including components of metabolic syndrome. Potential pathogenic factors that link UA to the development of type 2 diabetes include the following: endothelial dysfunction, impaired nitric oxide synthesis, oxidative stress, and subclinical inflammation. These factors are expected to lead to insulin resistance and subsequent carbohydrate metabolism abnormalities [12,13]. The primary causes of increased UA serum levels remain unclear but may include diet-related factors, such as excessive intakes of fructose and products containing purines [14,15].

The relationships between elevated levels of uric acid and gestational diabetes mellitus (GDM) and the development of type 2 diabetes in women with previous gestational diabetes mellitus (pGDM) have been poorly investigated. Single, prospective studies of this problem have revealed that an increased UA level in the first half of pregnancy multiplies the risks of GDM and pre-eclampsia, although not all authors have confirmed these observations [16–18]. A limited number of studies conducted with relatively small groups of women with pGDM, typically shortly after delivery, indicate that these women exhibit higher levels of UA than women without a history of GDM [19–21].

Thus, it seems worthwhile to test the hypothesis that an increased serum uric acid level is a missing link between pGDM and the development of type 2 diabetes later in life.

## Aim

The aim of the study was to evaluate the uric acid levels of women from a central-European population who had been diagnosed with pGDM several years previously and to compare these levels with the current nutritional status and carbohydrate metabolism of this population.

## Research Design and Methods

The study group consisted of 199 women who had given birth to their children within the past 5–12 years and had been diagnosed with GDM based on oral glucose tolerance tests (OGTTs) conducted during pregnancy (pGDM group). Anti-glutamic acid decarboxylase antibodies (anti-GAD) were negative in all the studied women. The control group comprised 50 women of comparable age who had given birth during the same period of time and for whom GDM had been ruled out based on the results of OGTTs during pregnancy, i.e., the C group. All studied women were Caucasian and lived in West Pomerania region in Poland. This study was approved by the Bioethics Committee of the Pomeranian Medical University in Szczecin, and written informed consent was obtained. It was a retrospective cohort study.

All study participants were interviewed to gather information about the number and course of their pregnancies. The physical examination included the following: measurements of height and weight and calculation of the body mass index [BMI (kg/m^2^)], measurements of the waist and hip circumferences and the calculation of the waist-hip ratio (WHR), blood pressure measurement according to the JNC 7 recommendations [22]. Body fat was measured using a Tanita SC-330S bioimpedance body composition analyzer (Tanita Corporation, Tokyo, Japan). An OGTT was performed for each woman and included analyses of the glucose levels (enzymatic method, Cormay SA, Warsaw, Poland) and the insulin levels (IRMA method, BioSource Europe SA, Nivelles, Belgium) at 0, 60 and 120 min. of the test. The baseline glucose and insulin levels were used to calculate the insulin resistance index (HOMA-IR) and β-cell function (HOMA-%B; the HOMA Calculator Software, v2.2.2) [23]. Glycated hemoglobin (HbA1c) was measured using the HPLC technique (Bio-Rad Laboratories, München, Germany). Creatinine levels were measured using the kinetic alkaline picrate method (Integra kit, Roche, Switzerland). The glomerular filtration rate (GFR) was estimated based on the Chronic Kidney Disease Epidemiology Collaboration (CKD-EPI). The study included only women with normal serum creatinine levels (<106 μmol/L). The uric acid and blood lipid levels were also measured (enzymatic-colorimetric method, Roche Diagnostics, Mannheim, Germany). Based on the results of the OGTTs and in accordance with the WHO guidelines, the study group was divided into the following subgroups: normal glucose tolerance (NGT), impaired fasting glucose (IFG), impaired glucose tolerance (IGT), and diabetes mellitus (DM) [24].

STATISTICA version 7.1 software (StatSoft Inc., Tulsa, OK, USA) was used for the database management and statistical analysis. The Mann-Whitney test and chi-square test were used to compare the continuous and nominal variables, respectively. The correlations between the continuous variables in each group were analyzed using Spearman’s rank correlation coefficients (Rs). Logarithmic transformations were applied for variables with non-normal distributions. P values <0.05 were considered statistically significant.

## Results

There were no differences between the pGDM group and the control group in terms of age (means 38.4 and 36.8 years), number of childbirths, time from indexed pregnancy (means 7.4 and 7.8 years), pre-pregnancy BMI, current anthropometric parameters (BMI and WHR), amount of body fat, non-adipose tissue, or blood pressure. In the women with pGDM, the mean HbA1c, fasting glucose, and glucose levels at 60 and 120 minutes of the OGTT were significantly higher than those in the controls but were still within the normal ranges (Table 1).

**Table 1:** Characteristics of the studied groups.

PARAMETER	pGDM (n = 199)	C (n = 50)	p
Age, years	38.4±6.6	36.8±5.6	NS
Time since index pregnancy, years	7.4±0.7	7.8±1.0	NS
BMI before pregnancy, kg/m^2^	22.4±3.4	22.5±4.1	NS
BMI, kg/m^2^	25.5±5.6	25.4±5.0	NS
WHR	0.86±0.09	0.84±0.07	NS
Adipose tissue mass, kg	21.9±10.2	22.6±11.1	NS
Adipose tissue mass, %	31.2±7.9	31.1±8.9	NS
Lean body mass, kg	45.5±6.1	46.1±4.8	NS
Systolic blood pressure, mm Hg	123.2±17.3	118.8±14.8	NS
Diastolic blood pressure, mm Hg	81.4±12.0	79.0±9.9	NS
HbA1c, %	5.6±0.4	5.4±0.4	<0.01
HbA1c, mmol/mol	37.7±4.4	35.5±4.4	<0.01
Glucose 0’, mmol/L	5.3±0.7	4.9±0.6	<0.0001
Glucose 60’, mmol/L	8.3±2.6	5.8±1.9	<0.0001
Glucose 120’, mmol/L	6.4±2.2	4.8±1.1	<0.0001
Insulin 0’, μIU/mL	13.7±8.7	13.7±8.5	NS
Insulin 60’, μIU/mL	106.8±62.6	83.8±41.1	<0.05
Insulin 120’, μIU/mL	74.6±58.7	47.5±30.6	<0.01
HOMA-IR	1.76±1.05	1.73±1.03	NS
HOMA-%B	125.4±52.7	145.7±49.2	<0.01
Uric acid, μmol/L	258.1±58.3	230.2±50.0	<0.005
Total cholesterol, mmol/L	4.93±1.0	4.99±0.88	NS
HDL-cholesterol, mmol/L	1.78±0.47	1.71±0.47	NS
LDL-cholesterol, mmol/L	2.68±0.93	2.79±0.8	NS
Triglycerides, mmol/L	1.11±0.69	1.06±0.97	NS
Creatinine, μmol/L	58.3±9.7	56.6±9.7	NS
eGFR CKD-EPI, mL/min.	110.2±12.1	115.1±12.0	NS

The pGDM group was characterized by significantly higher insulin levels at 60 and 120 min. of the OGTT, significantly lower insulin secretion as measured with the HOMA-%B score, and comparable insulin resistance parameters. No significant differences in lipid levels or renal function were found. The uric acid levels were significantly higher in the pGDM group (258.1±58.3 vs 230.2±50.0 μmol/L, p<0.005, Table 1).

**Fig 1:**
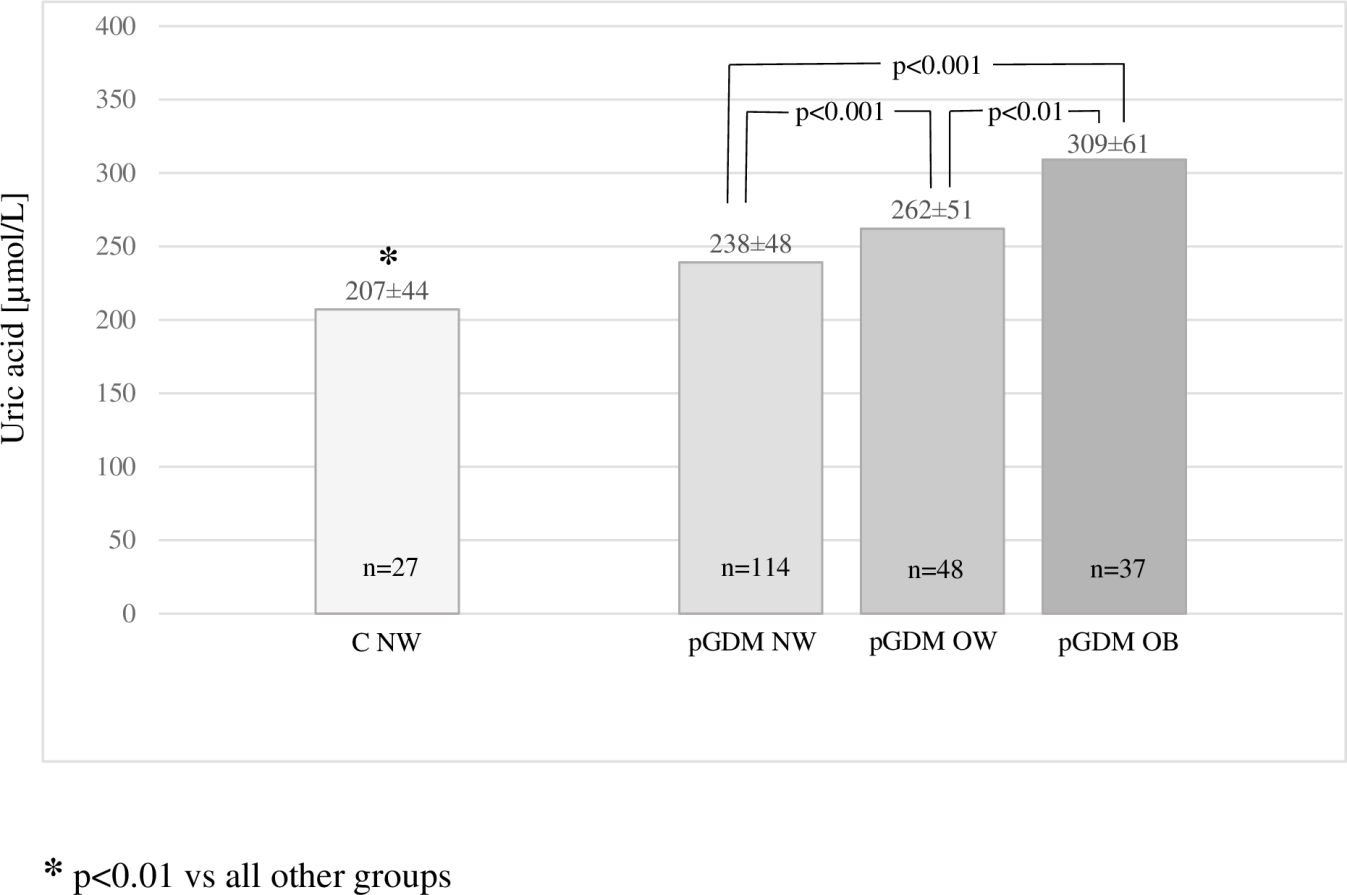
UA levels according to BMI status.

**Fig 2:**
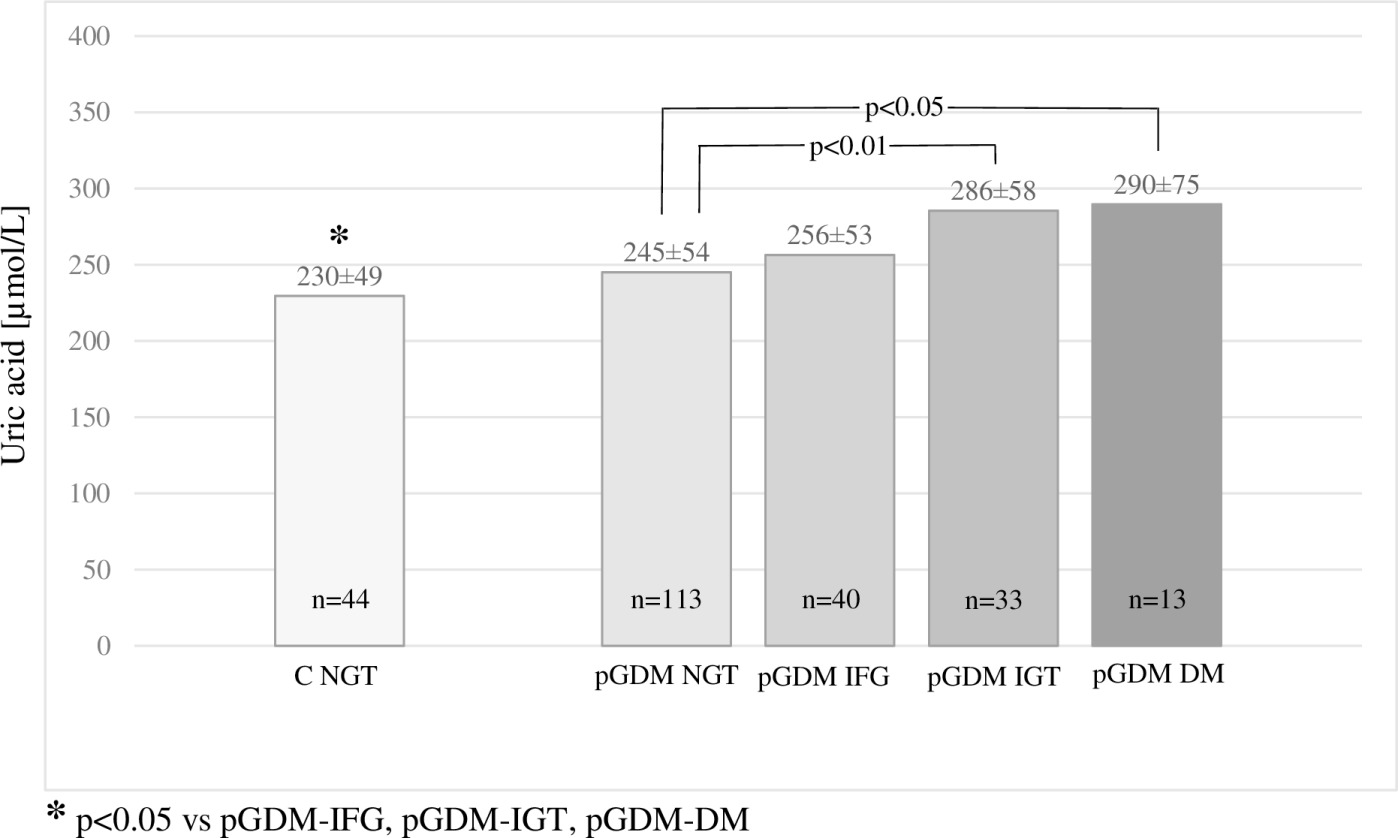
UA levels according to OGTT results.

In the pGDM group, the percentages of normal body weight (NW), overweight (OW), and obesity (OB) were 57.3%, 24.1% and 18.6%, respectively, and these percentages were comparable to those in the control group (54.0%, 28.0% and 18.0%, respectively). Abnormal glucose levels based on the OGTT results were observed in the pGDM group significantly more often than in the control group (43.2 vs 12.0%, respectively, p<0.001), and IFG was identified in 20.1%, IGT in 16.6%, and diabetes mellitus in 6.5% of the women. Some of the women in the control group were diagnosed with IFG (10.0%) and IGT (2.0%), but no diabetes mellitus was observed in this group. In the pGDM group, the UA levels increased with overweight, obesity, and the severity of carbohydrate metabolism disorders (Figs 1 and 2).

We observed strong positive correlations between UA and the following anthropometric parameters: BMI before pregnancy complicated by GDM, current BMI, body weight, WHR, and adipose tissue mass. Positive correlations were also observed between UA and the following metabolic parameters: HbA1c, glucose levels at all points of the OGTT, fasting insulin and insulin at 120 min of the OGTT, HOMA-IR, lipids, and serum creatinine. A negative correlation of UA level with HDL cholesterol was also observed. Multivariate analysis revealed significant correlations of UA level with BMI (β = 0.38, 95% CI 0.25–0.51, p<0.0001), creatinine level (β = 0.23, 95% CI 0.11–0.35, p<0.0005), triglycerides (β = 0.20, 95% CI 0.07–0.33, p<0.005) and family history of diabetes (β = 0.13, 95% CI 0.01–0.25, p<0.05). In logistic regression analysis, the association between higher UA level (defined as value ≥297 μmol/L) and presence of any carbohydrate metabolism disorder (IFG, IGT or diabetes) was statistically significant (odds ratio 3.62 [95% CI 1.8–7.3], p<0.001).

To exclude the influences of abnormal body weight and hyperglycemia on UA levels, an additional analysis involving only the group of women with normal body weights and normal glucose tolerances (NW+NGT) was performed. These criteria were met by 79 women from the pGDM group (39.7%) and 24 women from the control group (48.0%) (NS). There were no differences in the anthropometric parameters between these subgroups. In the NGT+NW subgroup of pGDM women, the UA levels were significantly higher (234.7±47.6 vs 208.2±47.6 μmol/L, p<0.05), and the glucose level at 60 min. of the OGTT and HDL cholesterol levels were also significantly higher. The insulin level at 60 min. and the glucose level at 120 min. of the OGTT were slightly higher in this group (the differences were near the limit of statistical significance; Table 2).

**Table 2:** Characteristics of the NGT+NW subgroups.

PARAMETER	pGDM NGT+NW (n = 79)	C NGT+NW (n = 24)	p
Age, years	37.0±6.2	35.1±5.2	NS
Time since index pregnancy, years	7.4±2.3	8.1±2.3	NS
BMI before pregnancy, kg/m^2^	20.7±2.0	20.2±1.5	NS
BMI, kg/m^2^	21.6±1.8	21.6±1.8	NS
WHR	0.83±0.05	0.82±0.06	NS
Adipose tissue mass, kg	15.5±4.2	14.6±4.3	NS
Adipose tissue mass, %	26.5±4.8	24.5±5.1	NS
Lean body mass, kg	42.2±2.5	43.7±3.3	NS
Systolic blood pressure, mm Hg	117.4±15.6	114.6±11.3	NS
Diastolic blood pressure, mm Hg	78.4±11.4	75.5±8.3	NS
HbA1c, %	5.5±0.4	5.4±0.4	NS
HbA1c, mmol/mol	36.6±4.4	35.5±4.4	NS
Glucose 0’, mmol/L	4.9±0.4	4.8±0.3	NS
Glucose 60’, mmol/L	6.7±1.5	5.5±1.4	<0.0005
Glucose 120’, mmol/L	5.2±1.0	4.8±1.1	NS
Insulin 0’, μIU/mL	11.1±7.7	10.9±2,6	NS
Insulin 60’, μIU/mL	98.9±62.6	74.2±34.4	NS
Insulin 120’, μIU/mL	61.4±52.3	47.2±26.5	NS
HOMA-IR	1.4±0.8	1.4±0.3	NS
HOMA-%B	126.2±46.0	132.8±24.6	NS
Uric acid, μmol/L	234.9±46.4	208.2±45.2	<0.05
Total cholesterol, mmol/L	5.0±1.1	4.7±0.5	NS
HDL-cholesterol, mmol/L	2.0±0.5	1.9±0.5	<0.05
LDL-cholesterol, mmol/L	2.6±1.0	2.5±0.5	NS
Triglycerides, mmol/L	0.9±0.4	0.8±0.4	NS
Creatinine, μmol/L	56.6±9.7	54.8±8.0	NS
eGFR CKD-EPI, mL/min.	112.2±11.7	116.2±9.2	NS

## Discussion

The number of papers that have been published thus far on the relationship between UA level and the risk of type 2 diabetes in women with pGDM is surprisingly scarce. The available studies have been conducted in small groups of women shortly after childbirth (0–4.5 years) and have mainly focused on other parameters, whereas UA levels have been investigated within a panel of disorders associated with metabolic syndrome [19–21,25]. The majority of the cited works indicate that women with pGDM exhibit elevated levels of UA.

Our study is the first to investigate the issue of UA in a large group of nearly 200 women and to be conducted several years after pregnancies complicated by GDM. Approximately 7.5 years after pregnancy complicated by diabetes mellitus, carbohydrate metabolism disorders were found in 43% of the subjects, which was 3.5-fold more frequent than in the women without histories of GDM. In the pGDM group, diabetes mellitus was diagnosed in 6.5% of the subjects, whereas in the control group, not a single case of DM was reported. The small percentage of women with diabetes probably resulted from the fact that the majority of the subjects had normal body weights (57.3%), while obesity was observed in 18.6% of the subjects. The slightly higher incidence of type 2 diabetes at 6 years after pGDM that was observed in another similar European population (9.2%) was probably related to the higher prevalence of obesity in that group of analyzed women (28.3%) [26].

The cause of the significantly higher incidence of carbohydrate metabolism disorders in women with pGDM is unclear because this group did not differ from the control group in terms of age, BMI (pre-pregnancy and current), or the amount of body fat. The lipid levels, insulin resistance indices, and blood pressure and renal values were almost identical. The differences were mainly found in the parameters of carbohydrate metabolism and uric acid levels. The women with pGDM exhibited higher fasting glucose, HbA1c, glucose and insulin levels during the OGTT and reduced insulin secretion as measured by the HOMA-%B index.

The UA levels in the women with pGDM were significantly higher than those of the controls (258.1 vs 230.2 mmol/L). The observed differences cannot be explained by differences in age, anthropometric and laboratory parameters of metabolic syndrome, or renal function. However, in the pGDM group these parameters were clearly linked with higher body weight and the severity of carbohydrate metabolism disturbances. The very strong correlation between uric acid level and BMI demonstrated by the multivariate analysis could explain the relationships with other parameters that increased in parallel with increases in BMI.

To rule out the potential influences of overweight, obesity and carbohydrate metabolism on the serum concentrations of uric acid, a subgroup of women with pGDM comprising only subjects with normal body weight (BMI<25 kg/m^2^) and normal glucose tolerance was distinguished. This subgroup was compared with controls who were selected in the same manner. The concentration of uric acid in the pGDM group was significantly higher but remained within the normal range (234.9 vs 208.2 μmol/L in the pGDM and control subgroups, respectively (Table 2)), and nearly identical anthropometric and laboratory insulin resistance parameters (i.e., fasting glucose and insulin, HOMA-IR, lipids, and HbA1c) were observed. The blood pressure and renal function of the two groups were very similar. The glucose level at 60 min of the OGTT was significantly higher in the pGDM group (6.7 vs 5.5 mmol/L), whereas the insulin level was slightly higher (98.9 vs 74.2 μIU/mL). These differences were no longer observed at 120 min of the OGTT. Because higher concentrations of UA could not have resulted from abnormal body weight or glucose intolerance, other factors responsible for this state need to be identified.

Studies from other authors have indicated that the serum uric acid level increases in parallel with the insulin level in response to a glucose stimulus, and this effect seems to be more pronounced in women [27–29]. This correlation has been observed both in subjects without diabetes and those with type 2 diabetes [29,30]. It seems that the increase in uric acid levels observed in the women with pGDM, including those with normal body weights and normal blood glucose levels, might be strongly related to glucose and glucose-stimulated insulin levels. In our study slight raise of uric acid concentration (still within normal range ≥297 μmol/L) was associated with a 3.64-fold increased risk of developing glucose metabolism disorders.

To our best knowledge, the present work is the first study to demonstrate higher levels of uric acid and increased glucose and insulin levels following glucose loads in women with a history of GDM, normal glucose tolerance and normal body weight.

If we assume that women predisposed to GDM exhibit persistent abnormal excessive glucose absorption after oral administration that is perhaps present pre-pregnancy, then these women may persistently respond to glucose stimuli with increased insulin secretion. Even a short-term increase in the glucose level due to stimulation by simple carbohydrates could lead to an increased glucose content in the glomerular filtrate and an increase in the amount of glucose reaching the proximal tubule. In turn, this process might lead to the activation of glucose transporters (GLUTs) that are present in the proximal tubule cells, including GLUT-9, which is also the uric acid transporter [31]. The increased glucose-stimulated activity of GLUT-9 could therefore be responsible for increased reabsorption of uric acid and the increased concentration of uric acid in the serum. The increase in the uric acid level could also result from an increased glucose-induced insulin level because insulin leads to the activation of the renin-angiotensin-aldosterone system and increased retention of UA in the proximal tubule [32]. Another primary abnormality, i.e., the genetically determined hyperactivity of GLUT-9 in the kidneys, also cannot be ruled out. In this case, serum glucose and insulin levels would be permanently increased even if they remained in the normal ranges.

A persistent increase in uric acid serum level, even if within the normal range, could produce negative effects. Such an increase could contribute to the formation of atherosclerotic plaques via the proliferation of smooth muscle cells in the vascular wall, increased macrophage migration into the vascular intima, and the stimulation of the synthesis of proinflammatory cytokines [33–35]. In higher concentrations, uric acid can damage and inhibit endothelial cell migration and can also interfere with the synthesis of NO to increase oxidative stress [36,37]. Through this mechanism, UA can initiate and sustain processes that lead to early atherogenesis, insulin resistance, and disorders of carbohydrate metabolism that are observed in women with pGDM.

The effects of uric acid on β-cells and insulin secretion have not been clearly explained. Results from previously cited clinical trials have indicated parallel increases in the levels of uric acid and insulin. It has been suggested that in subjects with higher concentrations of uric acid and increased insulin secretion who are observed in the early stage of type 2 diabetes, the depletion of β-cell function is more rapid [38]. Experimental in vitro studies in animals have demonstrated a toxic effect of uric acid on β-cells that leads to their impaired growth and apoptosis. This effect is thought to be associated with the activation of the nuclear factor NF-κB and oxidative stress [39,40].

Level of uric acid may increase in parallel with increased levels of insulin in response to a glucose stimulus. The reduced secretion of insulin could be caused by a gradual depletion of β-cell function that is related both to the chronic stimulation and the chronic toxic effects of uric acid on these cells. Conditions leading to increased insulin resistance, such as pregnancy and weight gain, could lead to a rapid decompensation and symptoms of carbohydrate metabolism disturbances, including gestational diabetes mellitus. Such defects could also contribute to the development of type 2 diabetes mellitus in women with a history of gestational diabetes mellitus, especially those who are overweight. More severe genetically determined disorders that are related to increased glucose/uric acid transporter activity in the kidneys or increased activities of the transporters responsible for excessive glucose absorption in the gastrointestinal tract could also explain the occurrence of GDM in women with normal body weight. This problem may be clarified by genetic studies of glucose and uric acid transporters in the kidneys (particularly GLUT-9) or in the gastrointestinal tracts of women with gestational diabetes mellitus, especially those who develop this despite having normal body weight and those with previous gestational diabetes mellitus who develop type 2 diabetes after pregnancy.

Our study has some limitations. For example, we did not consider the effects of diet, e.g., alcohol, fructose and high purine product consumption, on UA levels. Nevertheless, the group of almost 250 women included in our study was homogeneous in terms of ethnicity and the place of residence and followed a typical regional diet.

## Conclusions

Higher serum levels of uric acid may be associated with the development of type 2 diabetes in women with previous gestational diabetes also in these with normal body weights. The factors responsible for these associations needed to be clarified.

## Supporting Information

S1 File Data of the NTG+NW subgroups. (XLSX)
